# Socioeconomic differentials of trends in the prevalence and economic burden of chronic obstructive pulmonary disease in rural southwest China

**DOI:** 10.1186/s12889-023-15096-x

**Published:** 2023-01-20

**Authors:** Le Cai, Xu-Ming Wang, Lan Liu, Yi Zhao, Allison Rabkin Golden

**Affiliations:** 1 School of Public Health, Kunming Medical University, 1168 Yu Hua Street Chun Rong Road, Cheng Gong New City, Kunming, 650500 China; 2 The First Affiliated Hospital of Kunming Medical University, Kunming, China

**Keywords:** Chronic obstructive pulmonary disease, Economic burden, Temporal trend, Socioeconomic status, China

## Abstract

**Background:**

Chronic obstructive pulmonary disease (COPD) is a leading cause of mortality and morbidity, and imposes a substantial financial burden on society. However, few studies have examined the role of individual socioeconomic status (SES) in temporal trends of COPD prevalence and economic cost. This study aimed to uncover the changing prevalence and economic burden of COPD across socioeconomic gradients in rural southwest China.

**Methods:**

Data were collected from two cross-sectional health interviews and examination surveys administered 10 years apart among individuals aged ≥ 35 years in rural China. A prevalence-based cost-of-illness method was used to estimate the cost of COPD. The individual socioeconomic position (SEP) index was constructed using principal component analysis. Post-bronchodilator spirometry tests were performed for each participant.

**Results:**

From 2011 to 2021, the prevalence of COPD increased from 8.7% to 12.8% (*P* < 0.01), while the economic cost of COPD increased 1.9-fold. Unit hospital costs and outpatient costs increased 1.57–fold and 1.47–fold, while unit medication costs fell by 10.6%. Increasing prevalence was also observed when the data were stratified by sex, age, ethnicity, level of education, level of income, and SEP (*P* < 0.05). Men, ethnic minorities, and those with a lower educational level, lower income, or lower SEP had a higher prevalence of COPD than their counterparts both in 2011 and 2021 (*P* < 0.05). Unit outpatient costs and medication costs increased with patients’ SEP in both survey years (*P* < 0.05).

**Conclusions:**

The prevalence and economic costs of COPD increased substantially across all socioeconomic gradients in rural southwest China in the decade from 2011 and 2021. Future COPD prevention and management interventions as well as efforts to improve access to affordable COPD medication and treatment should focus in particular on ethnic minority and low SEP populations.

## Introduction

Chronic obstructive pulmonary disease (COPD), a chronic inflammatory respiratory disease, afflicts more than 380 million people worldwide as a leading cause of death and morbidity globally [1]. Beyond its heavy toll on human health, it also imposes a substantial economic burden [2], both when measured in terms of direct costs to healthcare systems and indirect costs to society [3].

China is both the world’s largest producer and largest consumer of tobacco products. COPD in China demands attention not only because it is highly prevalent across the nation, but its prevalence is also steadily rising due to China’s increased air pollution, rising rates of smoking, and aging population: COPD prevalence among those ≥ 40 years of age increased from 8.2% in 2002 to 13.6% in 2015, with higher rates observed in rural areas compared to urban environments [4–6]. In turn, COPD creates financial strain for patients, their families, and Chinese society as a whole [7, 8].

Traditionally, costs of disease have been estimated in terms of both direct and indirect costs. Numerous cost-of-illness studies over the years have been conducted on the estimated economic burden of COPD worldwide. However, the majority of these studies largely focused on direct costs, with relatively little emphasis on indirect economic consequences related to productivity loss [2]. In China, previous studies on COPD’s costs are limited and have focused on urban areas [9]; there is currently a lack of research investigating the economic costs of COPD in rural areas, where 70% of the population are farmers.

Previous research has identified key risk factors associated with increased risk of COPD, revealing tobacco smoking, exposure to passive smoking, indoor air pollution, and/or biological dusts, and history of asthma as well established major risk factors for COPD [1, 4, 6]. Additionally, several individual socioeconomic status (SES) factors are also associated with COPD prevalence: educational attainment, occupation, household income, and composite SES index are negatively correlated with COPD prevalence [10, 11], while low SES is associated with greater COPD morbidity. Yet, little research has been conducted to assess the role of individual SES in temporal trends of COPD prevalence, including in China.

In previous studies, the general approach to identify factors associated with costs of COPD has been to focus on individual demographic and clinical factors [2, 3, 9], and the association of SES factors with costs of COPD has rarely been examined. Further, the relationship between SES and increased economic burden of COPD over time remains unclear. In China, the association has especially received inadequate attention: these factors have rarely been examined in Chinese studies, and the association of SES and the changing dynamics of costs of COPD in China remains poorly understood.

In order to optimize the use of limited healthcare resources to best prevent and manage COPD, it is critical to understand the changing dynamics of COPD and its related costs in China, in particular in rural regions where the majority of COPD-related morbidity and mortality occurs [6, 12]. Indeed, the importance of understanding the financial challenge COPD poses in China is especially acute in the context of the growing societal and economic burden of the COVID-19 pandemic.

Thus, this study aimed to ascertain economic trends of COPD prevalence. Specifically, it employed a prevalence-based cost-of-illness methodology (including analysis of both direct and indirect costs) in order to uncover the change in societal economic burden of COPD in rural China from 2011 to 2021, and to examine how socioeconomic differences relate to the changing prevalence and costs of COPD over time.

## Methods

### Data sources and study population

Data were collected through two cycles of community-based, cross-sectional health interviews and surveys conducted in three rural areas of Yunnan Province across two time periods, separated by a decade: 2010–2011 and 2020–2021. Yunnan, a province of more than 48 million (as of 2020) in southwestern China, is one of China’s least developed provinces. With tobacco cultivated in more than a third of its counties (45 of 129 counties), its economy is driven by tobacco farming and consumption.

To select counties in Yunnan for health data collection, in both study years (2011 and 2021), all counties in the province were classified into three categories according to their wealth distribution (per capita GDP): low, medium, or high. One county was then randomly selected from each of these categories for a total of three counties selected in each of the two survey years. To choose study participants ≥ 35 years from the three selected rural counties, a consistent three-stage stratified random sampling selection process was then employed (greater detail on these sampling methods can be found in previous research [13]).

### Data collection and measurement

In both survey cycles, all consenting participants were interviewed in person by trained researchers using a structured, pre-tested survey to gather data on participant demographic characteristics (age, sex, ethnicity, household income, and education), smoking habits, respiratory symptoms, and family history of COPD. For participants who reported a previous diagnosis and/or treatment of COPD by a medical doctor, a detailed list of COPD-specific direct non-medical costs (including transportation and accommodation costs during hospital visits, and expenditures to hire caregivers), self-medication costs, and lost wages due to work absence were collected in addition. Information on the particular COPD treatments and medications used, inpatient hospitalization expenditures, and outpatient expenditures were also recorded from the medical records of the participants’ healthcare institutions.

Physicians performed spirometry in all participants using a portable data logging spirometer (CHESTHI-101, Japan) and measured pre- and post-bronchodilator spirometry following American Thoracic Society and European Respiratory Society (ATS/ERS) guidelines [14]. All tests were performed with the participant in a sitting position, and all participants rested for a minimum of 5 min as well as were guided through practice exhalations before each measurement was taken. Pre- and post-bronchodilator forced expiratory volume in one second (FEV1) and forced vital capacity (FVC) were recorded for all participants, and the ratio of these two measurements (FEV1: FVC) was calculated, with FEV1/FVC < 0.8 as the cutoff to perform post-bronchodilator spirometry. A minimum of three measurements were taken for all participants, and the highest value was considered the most accurate and used in our data analysis.

### Cost calculation

Total cost of COPD was calculated as the sum of two components: direct costs and indirect costs.

### Calculation of direct costs

All healthcare expenditures for treating COPD were defined as direct costs. They were determined by summing all cost categories in the preceding year, and subsequently delineated into direct medical costs and direct non-medical costs. Direct medical costs included expenses for outpatient visits, inpatient hospitalizations, and self-medications, while direct non-medical costs included transportation and accommodation costs for both the patient and their families when visiting healthcare providers.

### Calculation of indirect costs

Productivity losses due to COPD-related morbidity were defined as indirect costs. They were determined by multiplying the total number of days taken off work by both the patient and patient’s relatives by the mean daily gross income per person in both survey years. These means were determined using income data from the Yunnan Statistical Yearbook [15, 16]; rural household annual per capita net income was 3,952 RMB (US$613.20) in 2011 and 12,842 RMB (US$1992.60) in 2021.

Total costs incurred by the county were computed by multiplying the overall cost calculated for the participant group by the ratio of the entire county population divided by the participant population.

### Comparison of cost estimates across years

Constant 2021 US dollars were used to express all cost estimates. The estimated 2011 costs of COPD were inflated to 2021 constant Chinese Yuan (RMB) by multiplying by 1.039 based on the Consumer Price Index (CPI) [17] for 2011 and 2021 in China. Subsequently, using the 2021 exchange rate of $1 = 6.445 RMB, costs were converted to US dollars.

### Definitions

COPD was defined as post-bronchodilator FEV1/FVC < 70% in the present study, as defined by the Global Initiative for Chronic Obstructive Lung Disease (GOLD) [18].

All participants were classified into two education levels: illiterate (defined as the inability to either read or write a short simple statement about daily life among participants aged ≥ 15 years) or primary school (grade 1–6) or higher. Participants were also categorized by income into two annual household income groups, low or high, with the median annual household income of the participant group used as the cut-off point.

### Statistical analysis

All data analyses were conducted using SPSS 22.0 software. While descriptive statistics were computed with categorical variables presented as counts and percentages, continuous variables were presented as mean ± standard deviation (SD). To compare categorical variables between survey years, Chi-squared tests were computed. Wilcoxon rank sum or Kruskal–Wallis tests were employed for continuous variables. Prevalence of COPD was adjusted for age and sex by direct standardization to the 2020 Chinese population aged ≥ 35 years. Two-tailed *P* values were computed for statistical significance determinations, with *p* < 0.05 considered statistically significant.

To construct a composite index of individual socioeconomic position (SEP) on ethnicity, annual household income, and education— the three most correlated individual socioeconomic indicators from three socioeconomic dimensions based on an estimated Pearson correlation index > 0.5— principal component analysis (PCA) was performed. Bartlett’s test of sphericity was applied to determine whether the correlation between indicators was adequate, based on a criterion of *p* < 0.0001, while a criterion of Kaiser–Meyer–Olkin (KMO) statistic ≥ 0.7 was used to measure sample adequacy. Based on inspection of the scree plot, principal components were extracted, and Kaiser’s criterion of eigenvalues was ≥ 1.

## Results

The number of participants, all over the age of 35, invited to join in the two survey administrations was 8,400 in 2011, and 7,800 in 2021. Subsequently, 8,187 in 2011 and 7,572 in 2021 consented to participate, equating to an overall response rate of 97.5% and 97.1%, respectively.

Both the PCA with Bartlett’s test (*p* < 0.0001) and KMO Measure (0.70) results showed correlations between variables that were sufficiently large to perform the PCA. Only the first component with eigenvalues > 1 produced a three-component rotated solution that explained 50.2% of the total variance in the data. Consequently, only one component was retained to define the SEP index, and the SEP index was then further categorized into three levels: low, medium, and high.

Table 1 presents defining features of the participant population in the present study, separated by survey year. The proportion of men, ethnic minorities, participants with low annual household income, and participants with low SEP among the study participants did not differ between the two survey years (*P* > 0.05). In addition, women had both a lower level of education and lower SEP than males, a stable finding across the two survey years (*P* < 0.01). In contrast, the percent of participants of more than 40 years of age was greater in 2011 than in 2021 (*P* < 0.01) and the adult illiteracy rate decreased, from 30.5% in 2011 to 22.8% in 2021 (*P* < 0.01).

**Table 1:** General characteristics of the study population by survey year

Characteristics	Survey year					
	2011			2021		
	Male (*n* = 3960)	Female (*n* = 4227)	All (*n* = 8187)	Male (*n* = 3739)	Female (*n* = 3833)	All (*n* = 7572)
Age (%)						
35–39 years	428 (10.8)	340 (8.0)	768 (9.4)**	207 (5.5)	245 (6.4)	452 (6.0)
40–49 years	1102 (27.8)	1116 (26.4)	2218 (27.1)	760 (20.3)	821 (21.4)	1581 (20.9)
50–59 years	1001 (25.3)	1103 (26.1)	2104 (25.7)	1122 (30.0)	1036 (27.0)	2158 (28.5)
60–69 years	794 (20.1)	876 (20.7)	1670 (20.4)	863 (23.1)	911 (23.8)	1774 (23.4)
≥ 70 years	635 (16.0)	792 (18.7)	1427 (17.4)	787 (21.0)	820 (21.4)	1607 (21.2)
Ethnicity						
Han	2474 (62.5)	2534 (59.9)	5008 (61.2)	2035 (54.4)	2089 (54.5)	4124 (54.5)
Minority	1486 (37.5)	1693 (40.1)	3179 (38.8)	1704 (45.6)	1744 (45.5)	3448 (45.5)
Education level (%)						
Illiterate	711 (18.0)	1784 (42.2)**	2495 (30.5)**	667 (17.8)	1063 (27.7)**	1730 (22.8)
Primary (grade 1–6) or higher	3249 (82.0)	2443 (57.8)	5692 (69.5)	3072 (82.2)	2770 (72.3)	5842 (77.2)
Approximate annual household income						
Low	1923 (48.6)	2273 (53.8)*	4196 (51.3)	1877 (50.2)	1967 (51.3)	3844 (50.8)
High	2037 (51.4)	1954 (46.2)	3991 (48.7)	1862 (49.8)	1866 (48.7)	3728 (49.2)
SEP						
Low	654 (16.5)**	1546 (36.6)**	2200 (26.9)	964 (25.8)	1250 (32.6)**	2214 (29.2)
Medium	1479 (37.3)	1334 (31.6)	2813 (34.4)	1675 (44.8)	1566 (40.9)	3241 (42.8)
High	1827 (46.1)	1347 (31.9)	3174 (38.8)	1100 (29.4)	1017 (26.5)	2117 (28.0)
All	3960 (48.4)	4227 (51.6)	8187 (100.0)	3739 (49.4)	3833 (50.6)	7572 (100.0)

^*^p < 0.05

**p < 0.01

Age-adjusted prevalence of COPD in rural Yunnan, stratified by survey year and socioeconomic status, is presented in Table 2. From 2011 to 2021, the prevalence of COPD, as defined by spirometry, increased from 8.7% to 12.8% (*P* < 0.01). This increasing rate was also observed among the subgroups categorized by sex, age, ethnicity, education level, income level, and SEP (*P* < 0.05). In both 2011 and 2021, male and ethnic minority participants had a higher prevalence of COPD than female and Han ethnicity participants (*P* < 0.05). Further, lower education level, annual income, and SEP correlated with higher prevalence of COPD in both 2011 and 2021 (*P* < 0.05). Moreover, prevalence of COPD increased with age both in 2011 and 2021 (*P* < 0.01).

**Table 2:** Age-standardized prevalence of chronic obstructive pulmonary disease by survey year and socioeconomic status in rural Yunnan Province, China

Characteristics	2011	2021
	*n* (%)	*n* (%)
Sex		
Male	380 (9.9)*	581 (15.7)**
Female	376 (8.1)	373 (9.5)*
Age		
35–39 years	35 (4.4)**	21 (4.6)**
40–49 years	155 (6.8)	130 (8.1)
50–59 years	151 (7.5)	206 (9.6)
60–69 years	222 (13.1)	258 (14.3)
≥ 70 years	193 (13.8)	339 (21.2)
Ethnicity		
Han	366 (7.9)*	436 (10.4)*
Minority	390 (11.6)	518 (15.1)*
Level of education		
Illiterate	287 (10.8)**	241 (13.7)*
Primary (grade 1–6) or higher	469 (9.2)	713 (12.3)**
Approximate annual household income		
Low	503 (10.6)**	541 (14.4)**
High	253 (8.7)	413 (10.8)*
SEP		
Low	229 (10.8)	330 (14.5)**
Medium	320 (9.5)	398 (12.4)*
High	207 (8.2)	226 (10.6)*
All	756 (8.7)**	954 (12.8)

^*^p < 0.05

**p < 0.01

Table 3 outlines cost components of COPD data, separated by SEP and survey cycle. Total COPD-related costs were $83.6 million in 2011 and $159.2 million in 2021, representing a 1.9-fold increase over the ten-year study period. Unit hospital costs and outpatient costs also increased 1.57–fold and 1.47–fold, respectively, while unit medication costs fell by 10.6%. The largest percent of COPD-related costs— accounting for 97.2% in 2011 and 97.6% in 2021— were direct costs. Among these direct costs, hospital costs were the principal driver of direct medical expenditures as they made up more than half of total direct costs in both 2011 and 2021 (53.4% of the total direct costs in 2011 and 61.3% in 2021). Unit outpatient costs as well as medication costs increased in tandem with SEP in both survey cycles (*P* < 0.05).

**Table 3:** Cost of illness of chronic obstructive pulmonary disease (in USD) by survey year and socioeconomic position in rural Yunnan Province, China

Cost components	2011						2021					
	Unit cost				Total	% of total cost of illness	Unit cost				Total	% of total cost of illness
	Low SEP	Medium SEP	High SEP	All			Low SEP	Medium SEP	High SEP	All		
Direct medical costs	1041.5	1448.5	1609.9*	1340.2	113,188,164.6	89.8	1477.5	2458.9	2517.1*	1861.0**	231,416,479.6	93.7
Outpatient visits	109.0	242.1	376.3*	225.7**	19,063,228.4	15.1	319.6	829.1	820.3*	328.8	48,340,794.3	20.0
Hospitalizations	744.8	928.1	656.3	797.2**	67,333,938.5	53.4	962.0	1299.5	1361.5	1248.8	147,819,994.3	61.3
Medications	187.7	278.3	577.3*	317.2*	26,790,997.6	21.2	195.9	330.3	335.3*	283.5	35,255,690.9	14.6
Direct non-medical costs	104.2	167.8	201.0	152.6	12,889,662.1	10.2	80.4	80.8	71.1	78.4**	9,745,334.4	4.0
Direct costs	1145.7	1616.3	1810.9*	1492.8**	126,077,826.7	97.2	1557.9	2539.7	2588.2	1939.4	241,161,814.0	97.6
Indirect costs	36.5	44.0	51.7	43.6	3,679,765.3	2.9	50.7	47.5	43.6	47.5	5,903,924.6	2.4
Total cost of illness	1182.2	1660.3	1862.6**	1536.3	129,757,592.0	100.0	1608.6	2587.2	2631.8	1986.9**	247,065,738.6	100.0

^*^p < 0.05

**p < 0.01

## Discussion

Both the prevalence and the economic burden of COPD rose markedly over the ten-year study period, with significant socioeconomic differences in temporal trends for the observed rate and substantial costs to the rural southwest Chinese adult population.

The overall COPD prevalence rate observed in the study also exceeded prevalence rates observed in both other rural Chinese regions as well as other low- and middle-income countries [4, 7, 11]. Additionally, the present study determined men have a higher prevalence of COPD than women, a result that aligns with COPD sex differences that have been previously documented worldwide [1, 18]. Moreover, the data revealed that prevalence of COPD has increased considerably in both men and women in rural southwest China over the past decade, according with prior trends observed in China and globally [1, 12]. However, the notably increasing trend in prevalence of COPD was greater among men than in women. This may result from the significantly higher prevalence of current smokers among men versus women (64.9% vs. 1.5%) during the ten-year study period. In this way, our findings underscore the significant, and growing, public health challenge COPD presents to morbidity in rural southwest China.

In this study, COPD was more prevalent in ethnic minority populations, with ethnic minority participants experiencing higher prevalence of COPD than Han ethnicity counterparts in both 2011and 2021. Furthermore, the results of the present study also indicate that ethnicity is additionally correlated with temporal trends of COPD in rural southwest China: across all ethnic groups assessed during the ten-year study period, COPD levels increased, with ethnic minority participants experiencing both a higher prevalence of COPD than Han ethnicity participants as well as a greater increase over the study period than their Han counterparts. This may result from differing tobacco smoking habits between Han ethnicity and some ethnic minority groups, as tobacco smoking is a well-documented major risk factor for COPD [18, 19]: ethnic minority participants had higher overall prevalence of both smoking and passive smoking than Han ethnicity participants (39.8% and 33.1% vs. 36.8% and 22.8%), and an increasing rate of smoking was observed only in the ethnic minority participant population over the study period (39.8% in 2021 vs. 35.4% in 2011). The results thus suggest that COPD prevention and control efforts paired with interventions to reduce tobacco smoking are especially needed, and would be particularly impactful, in rural China’s minority communities.

There is growing evidence that low educational attainment and low income are associated with higher odds of having COPD [10, 11]. Our results are consistent with these studies, as participants with a high educational level and high income were less likely to suffer from COPD than their less educated and lower income counterparts in both 2011 and 2021. Moreover, high individual SEP had a negative association with prevalence of COPD, potentially because those with higher SEP tend to have a healthier lifestyle. This outcome demonstrates socioeconomic status correlates with COPD prevalence in rural China and accords with previous research conducted across high-, low-, and middle-income countries [10, 11, 20]. In addition, while the data indicated that prevalence of COPD increased across all education, income, and SEP categories over the study period, the relative increase of COPD prevalence was greater among low-income and low SEP participants. This possibly results from differences in health behaviors as well as differing environmental exposures across SES [20]. The findings thereby underscore that to head off an emerging epidemic in low-income and low-SEP populations, improvement in early detection and prevention of COPD is necessary.

The results show that direct and total economic costs of COPD increased between 2011 and 2021. Among recorded direct costs of COPD, while direct medical costs increased, direct non-medical costs (comprised of transportation and accommodation expenses for both the patient and their accompanying family members when visiting healthcare providers) decreased. The decreasing direct non-medical costs observed may result from recent government initiatives to “equalize access to public services and provide all with basic health care” that may have successfully improved access to affordable health services in rural populations.

Indirect costs represented a small share of total costs and thus did not greatly impact the total calculation of costs of COPD in our study in both 2011 and 2021. Moreover, indirect costs of COPD remained stable across 2011 and 2021, accounting for 2.4% and 2.9% of total costs of COPD in the two survey years, respectively. Previous studies have found that lost productivity due to COPD had a particularly high impact on the economy [2]. Our results somewhat differ from this conclusion, perhaps due to the fact that expense of productivity loss due to premature mortality was not included in indirect costs in our study. Inclusion of more COPD-related cost categories may result in higher estimated indirect costs.

In both 2011 and 2021, direct medical costs comprised the largest component of overall COPD-related expenses. Inpatient hospitalization costs made up the largest proportion of costs of these direct expenditures (> 53% of total direct costs). This finding accords with studies conducted in other areas of China [9, 21] as well as in Malaysia [22]. While direct medical costs increased substantially over the ten-year study period, in spite of the fact that a significant decrease in the average length of hospital stay was observed (18.4 days in 2011 vs. 12.3 days in 2021), the largest increases were in COPD hospitalization costs. Specifically, the per capita hospitalization costs of COPD were $797.20 in 2011 and $1248.80 in 2021, accounting for 130.0% and 90.5% of the local average annual household income in 2011 and 2021, respectively, and clearly indicating hospital expenditures impose a heavy financial burden on rural Chinese households. Previous studies in China and the United States also revealed an increase in COPD hospitalization costs over time [23, 24]. However, in contrast to these rising hospitalization costs, COPD medication costs decreased over the ten-year study period in the present study.

The present research also uncovered that individual SEP was a strong determinant of total costs of COPD. Namely, in both survey years, COPD patients with high SEP were more likely to have higher outpatient costs and medication costs than their low SEP counterparts, likely resulting from the fact that high SEP enables greater utilization of health services and increases financial access to and affordability of medication. Indeed, unaffordability and poor access to effective COPD medications remains a major barrier to quality of life of individuals with COPD in vulnerable communities, as the economic impact of chronic diseases has adversely and disproportionately affected low-income populations in the developing world [20, 25]. The findings thus highlight that, when determining allocation of financial resources, COPD treatment and management programs in rural China should give greater consideration to SEP.

Our study indicated that direct, indirect, and total economic costs of COPD increased across all SEP categories over the study period. However, the relative increase of inpatient hospitalization costs and total economic costs of COPD was greater among high SEP participants. This is possibly because those with high SEP have greater access to healthcare as well as greater ability to afford medication in the surveyed communities. The inverse association of individual SEP with costs of COPD suggests that in this study region, critical consideration should be given to those with low SEP when determining appropriation of financial resources in COPD treatment programs.

The present study is limited in two ways. First, recorded self-reported direct costs of COPD were based on participant recall, and may therefore be subject to bias. Second, costs resulting from premature death due to COPD were not included in total costs. Thus, the study likely underestimated the full costs of COPD.

## Conclusions

In sum, this study provides a comprehensive morbidity and cost analysis of COPD using a prevalence-based cost-of-illness methodology in a large representative sample. This study reveals an emerging COPD epidemic and an associated significant, and growing, economic burden in rural southwest China over the past decade, with both morbidity and economic costs substantially rising in the decade from 2011 and 2021. This indicates that COPD has become a major public health challenge in terms of both morbidity and economic burden in rural southwest China. In addition, this research uncovered the disparate impact of COPD on ethnic minorities and those with low SEP. In this way, the findings underscore an urgent need for improvement in early detection and prevention of COPD to head off an emerging epidemic and reduce the economic burden of COPD in rural southwest China, and suggest that future efforts to prevent and manage COPD and expand access to affordable care should focus on ethnic minority and low SEP populations.

## Data Availability

The datasets used and/or analyzed during the current study is available from the corresponding author on reasonable request.

## Abbreviations

ATS: American Thoracic Society
COPD: Chronic obstructive pulmonary disease
CPI: Consumer price index
ERS: European respiratory society
FEV1: Pre- and post-bronchodilator forced expiratory volume in one second
FVC: Forced vital capacity
GDP: Gross domestic product
GOLD: Chronic obstructive lung disease
KMO: Kaiser–Meyer–Olkin
PCA: Principal component analysis
SD: Standard deviation
SEP: Socioeconomic position
SES: Socioeconomic status
